# Comparative Evaluation of Centrifugation Speed and Its Impact on Diagnostic Accuracy

**DOI:** 10.1002/jcla.70120

**Published:** 2025-10-09

**Authors:** Mohammed A. Jeraiby, Jobran M. Moshi, Sajad A. Dar, Mahmoud M. Habibullah, Mjery Yahia, Saif Elden B. Abdalla, Raheem M. Balkhtab, Renad A. Saigh, Nahlah S. burayk

**Affiliations:** ^1^ Department of Basic Medical Science, College of Medicine Jazan University Jazan Saudi Arabia; ^2^ Department of Medical Laboratory Technology, College of Nursing and Health Science Jazan University Jazan Saudi Arabia; ^3^ Department of Nursing, College of Nursing and Health Science Jazan University Jazan Saudi Arabia; ^4^ Department of Laboratory Jazan University Hospital Jazan Saudi Arabia

**Keywords:** centrifugation speed, clinical laboratory testing, diagnostic accuracy, hemolysis rate

## Abstract

**Introduction:**

Timely laboratory results are essential for effective clinical decision‐making, with turnaround time (TAT) serving as a key performance indicator. Pre‐analytical steps like centrifugation significantly impact TAT. This study investigates whether a shorter, high‐speed centrifugation can reduce TAT while maintaining the analytical accuracy required for routine diagnostic testing.

**Methodology:**

This comparative experimental study analyzed blood samples from patients using plasma and serum tubes. Two centrifugation methods (A: (routine) 10 min at 3200 *g*; B: (faster) 5 min at 4000 *g*) were compared across chemistry, immunochemistry, coagulation, and interference indices.

**Results:**

Most analytes showed no significant difference between the two methods, indicating strong analytical agreement. However, significant differences were observed in sodium (*p* = 0.003), CKMB (*p* = 0.044), ALP (*p* = 0.001), ALT (*p* = 0.011), FRT4 (*p* < 0.001), PT (*p* = 0.008), and INR (*p* = 0.003). Despite these differences, all values remained within clinically acceptable ranges. Hemolysis rates decreased notably under the high‐speed method, dropping from 10.4% to 5.1%. Bias analysis across tube types revealed greater variability for CKMB and ALT under the high‐speed method (bias = 2.76 and –2.92; CVs = 68.8% and 84.16%, respectively).

**Conclusion:**

High‐speed centrifugation significantly reduces processing time without compromising clinical reliability. Despite minor analyte differences, all values ramined within acceptable clinical ranges. Furthermore, the observed reduction in hemolysis rates strengthens the case for adopting the high‐speed method as a reliable, efficient, and clinically safe alternative for routine laboratory workflows.

## Graphical abstract

This study evaluates whether high‐speed centrifugation (5 min at 4000 *g*) can effectively reduce laboratory turnaround time (TAT) without compromising diagnostic accuracy. Results showed that most analytes remained clinically reliable, hemolysis rates were significantly reduced, and the faster method proved to be a safe and efficient alternative for routine blood testing.

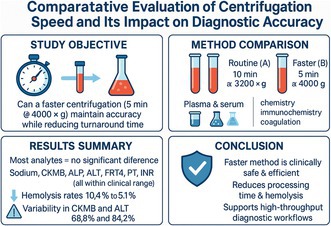

## 1 Introduction

Laboratory testing plays an important role in modern clinical practice, which guides the diagnosis of diseases, monitors their progression, and evaluates the effectiveness of the treatment [1]. Among the critical performance indicators for laboratory services, the turnaround time (TAT) is a key parameter that directly impacts patient care, clinical workflow, and hospital efficiency [2]. If there is a delay in the TAT, it not only hinders timely clinical decision‐making but may also contribute to prolonged hospital stays, which increase healthcare costs and reduce patient satisfaction. The pre‐analytical phase, which consists of specimen collection, its labeling, transport, and centrifugation, contributes significantly to the overall TAT, with centrifugation being one of the most time‐consuming steps [3].

Traditionally, the laboratories follow the standard centrifugation protocols which balance the adequate plasma or serum separation with the minimal risk of hemolysis and measure the analyte accurately [4]. As the healthcare systems worldwide experience the rising patient volumes and the growing demand for rapid diagnostics, particularly in emergency and outpatient settings, there is an increasing push to optimize pre‐analytical workflows, specifically through alternative centrifugation protocols. One such approach involves reducing centrifugation time while increasing relative centrifugal force (RCF), thereby accelerating sample processing without compromising the quality of laboratory results [5]. The previous researches have reported that the modifications in the centrifugation parameters may offer the time‐saving benefits without significantly affecting the stability or integrity of many biochemical analytes [3]. However, these findings often depend on the sample type, analyte sensitivity, and the specific analyzer characteristics. Thus, any proposed protocol shift requires thorough validation against conventional standards to ensure analytical accuracy, precision, and clinical equivalence. Additionally, concerns about increased hemolysis, fibrin formation, or analyte drift with higher‐speed, shorter‐duration centrifugation must be carefully addressed. This study evaluates the effectiveness of an alternative centrifugation method—High‐speed centrifugation method (5 min at 4000 *g*) in comparison with the widely adopted routine centrifugation method (10 min at 3200 *g*) [6]. The primary objective of this study is to assess whether High‐speed centrifugation method can significantly reduce the overall centrifugation time and consequently the TAT without compromising the analytical reliability of routine laboratory tests. The analytes assessed in this comparison include commonly ordered blood chemistry markers such as electrolytes, liver enzymes, and coagulation profiles, which are critical for rapid clinical decisions in settings like emergency departments, ICUs, and preoperative evaluations. Moreover, the hemolysis index, sample clarity, and frequency of pre‐analytical errors serve as secondary indicators to evaluate the clinical acceptability of the modified protocol [7]. Given that hemolysis is one of the most common causes of sample rejection which leads to false biochemical values, maintaining low hemolysis rates remains a non‐negotiable requirement for adopting faster centrifugation techniques [8]. In the real‐world laboratory setting, if we implement a faster centrifugation protocol, it can significantly boost laboratory efficiency by halving processing time, which eases the peak‐hour congestion, and expediting result delivery which supports the global standards for rapid diagnostics and strategically enhancing patient care and healthcare system responsiveness. Thus, adopting a new centrifugation method requires strong evidence to confirm analytical reliability across diverse samples. Practical considerations like equipment compatibility and staff training must also be addressed to ensure successful implementation and maintain consistent laboratory performance. By addressing both time efficiency and test reliability, this research aspires to inform laboratory policy updates and promote evidence‐based quality improvement in clinical laboratory operations.

## 2 Methods

This comparative experimental study was conducted following approval from the Jazan University Ethics Committee (Approval Number: REC‐46/06/1284). Informed consent was obtained from all participants prior to enrollment. The study was designed to compare the effects of two centrifugation methods on laboratory results across clinical chemistry, immunochemistry, and coagulation assays. It was conducted at the Phlebotomy Services Unit within the Department of Laboratory Medicine, Jazan University Hospital. All procedures were performed by trained phlebotomists according to standardized protocols.

A total of 150 participants were enrolled in this study. For each participant, the following blood samples were collected: two lithium heparin tubes and two serum tubes without gel and clot activator (V‐PRO 2, ADVANCE MEDICAL.CO RIYADH‐KSA) for general chemistry and immunochemistry analysis. Two sodium citrate tubes (3.2%) (V‐PRO, ADVANCE MEDICAL.CO RIYADH‐KSA) for coagulation testing yielded approximately 900 samples in total. Samples were processed using two different centrifugation methods: A: centrifugation for 10 min at 3200 *g* and B: centrifugation for 5 min at 4000 *g*. All centrifugation was carried out using a UNIVERSAL 320 R refrigerated centrifuge (Hettich Zentrifugen, Germany). The rotor used was a swing‐out rotor. The samples were centrifuged at a controlled temperature of 19°C. The device has a maximum rotational speed of 16,000 rpm (rpm).

The appearance of serum or plasma was visually inspected for signs of interference by two highly qualified laboratory specialists. A red discoloration indicated hemolysis (categorized as mild, moderate, or severe), a turbid or creamy‐white color indicated lipemia, while yellowish discoloration suggested icteric. In addition to excluding clotted or fibrin‐containing specimens, we screened and rejected samples presenting other preanalytical issues in accordance with established laboratory protocols. These included improper or missing labeling, use of inappropriate tubes (e.g., incorrect tube type for the requested test), insufficient sample volume, and inadequate sample‐to‐anticoagulant ratio. Biochemistry tests were performed using the Beckman Coulter DXC 700 AU analyzer. Immunochemistry tests were performed using the Beckman Coulter DXI 600. Coagulation tests were carried out using the STAGO Compact analyzer.

Data were analyzed using SPSS software (29.0.0). Descriptive statistics were reported as mean ± standard deviation (SD) and interquartile range (IQR) for continuous variables. Paired sample t‐tests were used to compare analyte values between A (10 min at 3200 *g*) and B (5 min at 4000 *g*). Bland–Altman plots assessed agreement between protocols, with mean bias and 95% limits of agreement calculated for each analyte. Coefficient of variation (CV) was computed to evaluate precision between tube types (green‐top vs. yellow‐top). A *p*‐value of < 0.05 was considered statistically significant. Interference indices (hemolysis, lipemia, icterus) were evaluated using frequency counts and chi‐square tests where applicable to detect protocol‐related differences.

## 3 Results

**FIGURE 1:**
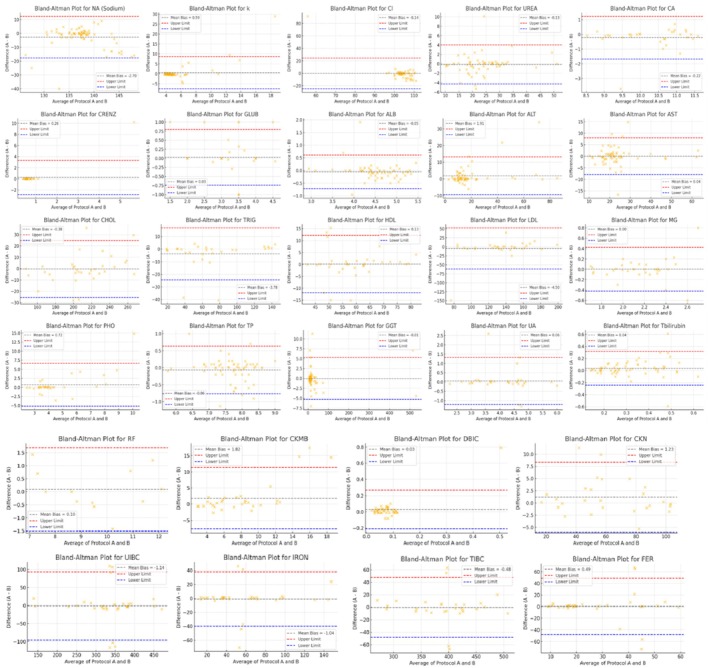
Bland–altman agreement analysis for the general chemistry analyte. Protocol A (Routine Centrifugation Method), Protocol B (High‐Speed Centrifugation Method).

**FIGURE 2:**
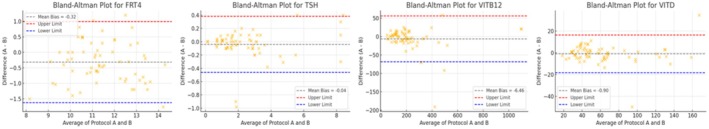
Bland–Altman agreement analysis for the immunochemistry analyte. Protocol A (Routine Centrifugation Method), Protocol B (High‐Speed Centrifugation Method).

**FIGURE 3:**
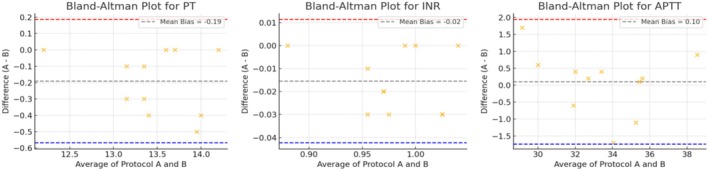
Bland–Altman agreement analysis for the coagulation analyte. Protocol A (Routine Centrifugation Method), Protocol B (High‐Speed Centrifugation Method).

To evaluate the analytical impact of centrifugation speed, we compared key laboratory analytes processed using the routine and high‐speed centrifugation methods. Most analytes showed no statistically significant differences, indicating strong analytical agreement and suggesting that the high‐speed method maintains clinical reliability. However, a few analytes demonstrated statistically significant deviations: sodium (Na, *p* = 0.003), CKMB (*p* = 0.044), ALP (*p* = 0.001), ALT (*p* = 0.011), FRT4 (*p* < 0.001), Table 1. Despite these differences, the mean values stayed within clinically acceptable ranges defined by internal quality standards and CLIA guidelines, suggesting no practical impact on clinical interpretation or patient care. Interference indices (lipemia, icterus, hemolysis) showed similar or reduced occurrences under the high‐speed method. Notably, hemolysis dropped from 10.4% to 5.1%, hinting at gentler handling or reduced exposure time minimizing red cell rupture. To further assess the influence of tube type on analyte performance, Table 2 provides a detailed comparison of results across two centrifugation methods using green‐top (lithium heparin) and yellow‐top (no additive) tubes. Overall, most analytes showed no statistically significant differences between tubes or methods, indicating consistent analytical performance across tube types. Notably, sodium (Na) exhibited a statistically significant difference (*p* = 0.003), with elevated levels in the yellow tube under the high‐speed method (140.51 ± 8.39) compared to the others. This discrepancy may reflect tube‐specific factors such as sample stability or the absence of additives in yellow tubes. However, despite this variation, sodium values remained within clinically acceptable limits. Other parameters, including potassium (K), ALT, AST, CKMB, FRT4, TSH, and lipid markers, did not show significant differences across tube types or methods. The minimal variation between the routine and high‐speed methods, regardless of the tube used, supports the robustness of the high‐speed method across different sample containers. Importantly, interference indices like hemolysis (HEM), icterus (ICT), and lipemia (LIP) remained low and comparable, with only a slight increase in hemolysis observed in yellow tubes under the high‐speed method. Figure 1 displays the Bland–Altman agreement plots for general chemistry analytes comparing the routine centrifugation method and the high‐speed centrifugation method. Most analytes demonstrate tight clustering around the zero line with the majority of differences falling within the 95% limits of agreement, indicating strong analytical consistency between the two methods. Sodium (Na) exhibits a slight negative bias of −2.70, while ALT and CKMB show mild downward biases (−1.91 and −4.50, respectively), consistent with earlier statistical findings. However, these differences remain within clinically acceptable ranges. Triglycerides (TRIG) show broader scatter with a bias of −3.78, but still fall within acceptable limits. In contrast, analytes such as albumin (ALB), total protein (TP), HDL, uric acid (UA), and chloride (Cl) display negligible bias, indicating near‐perfect agreement. Notably, GGT and creatinine (CRENZ) show more dispersed values, likely due to intrinsic variability, although without significant mean bias. Figure 2 shows Bland–Altman plots assessing agreement between the routine centrifugation method and the high‐speed centrifugation method for immunochemistry analytes. Overall, the plots demonstrate good agreement, with most data points falling within the 95% limits of agreement and clustering close to the zero‐bias line. FRT4 shows a mean bias of −0.32, while TSH reveals a minimal bias of −0.04, indicating near‐identical results between methods. These slight differences are clinically negligible, affirming the reliability of the high‐speed method. Vitamin D (VITD) has a slightly broader spread, with a mean bias of −0.90, yet the values remain within the allowable bias range for immunoassay‐based methods. However, despite the statistical shift, most values fall within the limits of agreement and remain clinically interpretable. In summary, the plots confirm that the high‐speed method maintains analytical comparability for immunochemistry testing. Figure 3 shows the Bland–Altman plots for three key coagulation analytes—Prothrombin Time (PT), International Normalized Ratio (INR), and Activated Partial Thromboplastin Time (APTT)—comparing the routine centrifugation method and the high‐speed centrifugation method. All three analytes show excellent analytical agreement with minimal bias and tight clustering of data points within the 95% limits of agreement. The PT plot demonstrates a slight mean bias of −0.19, indicating that values measured under the high‐speed method tend to be marginally lower than those from the routine method. This difference, however, is well within clinical tolerance and unlikely to influence therapeutic decisions. Similarly, APTT, with a mean bias of 0.10, shows slightly more dispersion than INR but remains within clinically acceptable limits, and no outliers breach the upper or lower agreement thresholds. Overall, these findings confirm that the high‐speed method maintains excellent analytical performance for coagulation studies. The negligible biases suggest that faster centrifugation does not affect the accuracy of coagulation parameters, thereby validating the high‐speed method for routine use in coagulation panels to reduce turnaround time without compromising result reliability.

**TABLE 1:** Side‐by‐side comparison of analyte results between the routine centrifugation method and the high‐speed centrifugation method.

Analyte	Routine centrifugation method		High‐speed centrifugation method		*p*
	Mean (SD)	IQR	Mean (SD)	IQR	
Na	136.24 (5.26)	3.35	138.94 (6.06)	3.65	**0.003** ^a^
k	5.72 (4.43)	0.8	5.13 (1.58)	0.97	0.263^a^
Cl	103.64 (3.15)	3	103.78 (12.83)	4.22	0.929^a^
ALB	4.50 (0.50)	0.52	4.55 (0.56)	0.6	0.231^a^
CKMB	8.90 (5.94)	4.96	7.08 (2.73)	3.58	**0.044** ^a^
HDL	59.42 (10.75)	12.2	59.29 (10.38)	12.76	0.902^a^
UIBC	332.98 (76.38)	89.98	334.11 (78.37)	93.41	0.896^a^
PHO	4.91 (3.07)	1.16	4.19 (1.40)	1.02	0.166^a^
ALP	76.57 (72.15)	18.94	81.73 (71.14)	21	**0.001** ^a^
ALT	20.28 (18.73)	8.5	18.37 (16.67)	7.33	**0.011** ^a^
IRON	57.88 (37.89)	41.48	58.92 (36.24)	42.39	0.765^a^
MG	2.15 (0.30)	0.31	2.15 (0.27)	0.37	0.955^a^
UA	4.13 (0.90)	1.26	4.07 (0.97)	1.44	0.610^a^
RF	9.62 (1.72)	2.8	9.52 (1.84)	2.6	0.692^a^
DBIC	0.09 (0.13)	0.05	0.06 (0.03)	0.06	0.114^a^
AST	23.33 (10.23)	7.09	23.29 (9.98)	6.59	0.930^a^
FER	28.29 (19.05)	27.43	27.80 (19.42)	21.2	0.909^a^
T‐ Bilirubin	0.34 (0.15)	0.23	0.30 (0.13)	0.19	0.057^a^
TP	7.64 (0.63)	0.73	7.70 (0.68)	0.8	0.161^a^
GGT	34.67 (92.53)	7.14	34.68 (92.38)	7.53	0.978^a^
CHOL	209.99 (34.76)	41	210.37 (29.99)	34.18	0.866^a^
CKN	62.17 (26.73)	39.35	60.95 (27.38)	43.96	0.093^a^
GLU	88.32 (38.73)	26.74	86.72 (38.88)	33.79	0.679^a^
TRIG	69.52 (40.89)	55.2	73.30 (39.05)	52.2	0.063^a^
UREA	23.63 (8.46)	9.95	23.77 (8.08)	8.46	0.635^a^
CA	10.19 (1.02)	1.5	10.41 (0.93)	1.38	0.100^a^
CRENZ	0.87 (1.57)	0.15	0.61 (0.14)	0.08	0.288^a^
GLUB	3.15 (0.54)	0.08	3.13 (0.59)	0.18	0.595^a^
LDL	132.97 (38.37)	35.75	137.47 (28.05)	36.25	0.405^a^
TIBC	392.55 (58.62)	61	393.03 (59.61)	67	0.915^a^
FRT4	11.25 (1.41)	1.69	11.57 (1.48)	2.34	**0.000** ^a^
TSH	2.46 (1.93)	1.76	2.50 (1.91)	1.8	0.145^a^
VITB12	222.29 (186.57)	112	228.75 (189.63)	134	0.097^a^
VITD	60.21 (33.95)	28.42	61.11 (34.04)	33.03	0.398^a^
PT	13.36 (0.53)	0.55	13.55 (0.58)	0.6	**0.008** ^a^
INR	0.97 (0.04)	0.05	0.99 (0.05)	0.05	**0.003** ^a^
APTT	33.51 (2.63)	3.2	33.41 (2.88)	3.45	0.731^a^
LIP (*N*)	4 (5.2%)		3 (3.8%)		**1.000** ^b^
ICT (*N*)	0 (0.0%)		0 (0.0%)		—
HEM (*N*)	8 (10.4%)		4 (5.1%)		**0.530** ^b^

*Note:* Significance of bold values indicates statistically significant *p*‐values when *p* < 0.05.

^a^ Independent sample *t*‐test.

^b^ Chi‐square test.

**TABLE 2:** Side‐by‐side comparison of analyte results between the routine centrifugation method and the high‐speed centrifugation method across tube types (Green‐top vs Yellow‐top).

	Routine centrifugation method		High‐speed centrifugation method		Sig. value
	Green tube	Yellow tube	Green tube	Yellow tube	
NA	136.55 (4.87)	136.24 (4.55)	137.31 (3.26)	140.51 (8.39)	**0.003** ^**a**^
k	5.24 (1.99)	5.64 (4.78)	5.27 (1.87)	6.20 (5.25)	0.662^a^
Cl	103.64 (2.80)	103.36 (3.38)	103.83 (3.37)	103.84 (16.25)	0.994^a^
ALB	4.50 (0.53)	4.51 (0.51)	4.50 (0.48)	4.48 (0.60)	0.997^a^
CKMB	8.83 (5.94)	8.37 (3.95)	7.69 (4.38)	10.46 (7.19)	0.471^a^
HDL	57.84 (10.44)	59.45 (10.32)	57.68 (10.16)	59.43 (11.30)	0.908^a^
UIBC	335.11 (79.50)	330.61 (80.66)	336.48 (71.74)	339.10 (79.57)	0.990^a^
PHO	5.02 (2.97)	4.86 (3.28)	4.14 (1.52)	5.36 (3.15)	0.531^a^
ALP	76.94 (67.76)	77.65 (69.03)	77.07 (61.35)	86.96 (71.18)	0.913^a^
ALT	19.59 (17.19)	20.92 (18.61)	20.86 (18.63)	17.94 (15.10)	0.860^a^
IRON	59.19 (36.29)	56.80 (37.16)	58.61 (36.31)	63.55 (40.26)	0.953^a^
MG	2.12 (0.27)	2.13 (0.32)	2.20 (0.48)	2.15 (0.23)	0.887^a^
UA	4.05 (0.88)	4.14 (0.82)	4.10 (0.88)	4.03 (0.97)	0.981^a^
RF	9.88 (1.97)	9.58 (1.88)	10.05 (1.68)	9.61 (1.82)	0.948^a^
DBIC	0.07 (0.03)	0.08 (0.03)	0.10 (0.16)	0.06 (0.03)	0.403^a^
AST	22.93 (9.20)	22.97 (9.73)	23.89 (9.24)	23.78 (9.79)	0.953^a^
FER	26.88 (18.84)	27.41 (19.50)	27.19 (18.92)	30.76 (21.02)	0.918^a^
T. Br	0.35 (0.16)	0.35 (0.16)	0.32 (0.16)	0.28 (0.11)	0.113^a^
TP	7.65 (0.62)	7.55 (0.62)	7.63 (0.61)	7.67 (0.74)	0.851^a^
GGT	32.40 (84.35)	33.22 (83.16)	32.77 (80.45)	34.02 (85.00)	1.000^a^
CHOL	205.44 (32.91)	209.69 (34.74)	205.30 (32.19)	210.46 (30.61)	0.934^a^
CKN	67.82 (31.42)	61.42 (25.62)	67.09 (31.21)	63.51 (25.49)	0.901^a^
GLU	90.21 (42.02)	94.36 (54.79)	84.04 (44.60)	94.15 (58.64)	0.787^a^
TRIG	72.33 (42.01)	79.54 (44.18)	71.09 (43.46)	78.71 (45.47)	0.902^a^
UREA	23.31 (8.33)	23.46 (8.25)	23.29 (8.07)	24.31 (8.65)	0.945^a^
CA	10.28 (0.96)	10.17 (1.10)	10.19 (0.98)	10.38 (0.92)	0.916^a^
CRENZ	0.65 (0.15)	0.65 (0.17)	0.62 (0.16)	1.01 (1.99)	0.359^a^
GLUB	3.15 (0.57)	3.23 (0.54)	3.09 (0.50)	3.21 (0.71)	0.681^a^
LDL	133.71 (29.56)	137.06 (28.37)	133.73 (28.58)	128.06 (41.20)	0.865^a^
TIBC	401.79 (53.33)	395.47 (61.79)	400.11 (51.79)	398.18 (61.14)	0.989^a^
FRT4	11.16 (1.36)	11.21 (1.36)	11.34 (1.39)	11.56 (1.42)	0.587^a^
TSH	2.77 (2.07)	2.50 (1.85)	2.72 (2.07)	2.52 (1.81)	0.909^a^
VITB12	214.63 (178.08)	215.51 (178.54)	230.72 (181.80)	209.12 (177.93)	0.952^a^
VITD	64.81 (34.92)	60.97 (35.27)	63.65 (33.43)	61.97 (38.01)	0.961^a^
LIP	0 (0.0%)	1 (2.63%)	2 (5.26%)	3 (7.90%)	0.993^b^
ICT	0 (0.0%)	0 (0.0%)	0 (0.0%)	0 (0.0%)	—
HEM	0 (0.0%)	0 (0.0%)	2 (5.26%)	3 (7.89%)	—

*Note:* Yellow tubes (no additive), green tube (lithium heparin). Significance of bold values indicates statistically significant *p*‐values when *p* < 0.05.

^b^ ANOVA.

^a^ Chi‐Square test.

Table 3 shows the mean bias and coefficient of variation (CV) for analytes assessed under the routine centrifugation method and the high‐speed centrifugation method. The results reinforce the analytical reliability of the faster centrifugation approach, with most analytes exhibiting minimal bias and acceptable precision. Significant bias was noted in Sodium (Na) (−1.76), yet both methods maintained low CVs (4.25% vs. 4.09%), indicating stable precision with a clinically acceptable bias that does not compromise electrolyte management. CKMB showed a positive bias of 1.82, with a marked reduction in variability under the high‐speed method (CV: 38.5% vs. 66.73%), suggesting improved measurement consistency despite the bias. Similarly, ALT (bias: +1.91) and LDL (−4.5) showed moderate differences but remained within quality control thresholds. Dramatic improvements in CV were seen for CRENZ (180.56%–23.79%), DBIC (136.25%–52.82%), and potassium (K) (77.34%–30.8%), highlighting significant gains in precision with the high‐speed method. While analytes like GGT and VITB12 maintained high CVs across both methods, the consistency in bias (near zero) supports their clinical reliability. Importantly, phosphorus (PHO) also showed improved CV (62.57%–33.34%) and minor bias (+0.72), further supporting the robustness of the high‐speed method. Overall, the faster centrifugation method demonstrated equivalent or superior precision across most analytes, with clinically tolerable biases, making it a viable alternative for routine diagnostics.

**TABLE 3:** Bias and coefficient of variation (CV) between the routine centrifugation method and the high‐speed centrifugation method.

Analyte	Bias (A—B)	CV—Protocol A (%)	CV—Protocol B (%)
NA (Sodium)	−1.76	4.25	4.09
k	0.59	77.34	30.8
Cl	−0.14	3.04	12.36
UREA	−0.13	35.78	34.01
CA	−0.22	9.98	8.89
CRENZ	0.26	180.56	23.79
GLUB	0.03	17.17	18.73
ALB	−0.05	11.19	12.26
ALT	1.91	92.37	90.76
AST	0.05	43.86	42.87
TIBC	−0.48	14.93	15.17
GGT	−0.01	266.9	266.38
TP	−0.06	8.23	8.88
DBIC	0.03	136.25	52.82
CKMB	1.82	66.73	38.5
UA	0.06	21.79	23.75
MG	0.0	13.75	12.42
RF	0.1	17.91	19.3
FER	0.49	67.32	69.86
IRON	−1.04	65.46	61.51
HDL	0.13	18.09	17.5
CHOL	−0.38	16.55	14.25
LDL	−4.5	28.86	20.41
TRIG	−3.78	58.82	53.27
CKN	1.23	43.0	44.93
FRT4	−0.32	12.5	12.81
TSH	−0.04	78.36	76.13
VITB12	−6.46	83.93	82.9
VITD	−0.9	56.38	55.7
PHO	0.72	62.57	33.34
UIBC	−1.14	22.94	23.46
Tbilirubin	0.04	44.14	44.43
PT	−0.19	3.95	4.26
INR	−0.02	4.48	4.66
APTT	0.1	7.83	8.62

Table 4 shows the inter‐tube bias (Yellow–Green) and coefficient of variation (CV) for each analyte under both centrifugation methods. This helps assess whether tube type (serum vs. heparin plasma) influences test consistency and whether this variation is impacted by centrifugation speed and duration. Under the routine centrifugation method, most biases were minimal. For example, sodium (Na) had a negligible bias of −0.31 and low CV (3.34%). However, ALT (bias = 1.34; CV = 88.95%), CKMB (bias = −0.45; CV = 47.16%), and GGT (bias = 0.81; CV = 250.36%) showed substantial variability between tube types. Under the high‐speed centrifugation method, biases generally remained acceptable, but variability in a few analytes increased. Notably, CKMB bias increased to 2.76 with CV rising to 68.8%, indicating tube‐related inconsistency under faster centrifugation. CRENZ showed a spike in CV (197.64%), likely due to outliers or instability in serum under high‐speed centrifugation. ALT bias reversed (−2.92) and CV remained high (84.16%), though within prior trends. Analytes such as TIBC, FRT4, calcium (CA), and total protein (TP) remained stable across methods and tubes, showing minimal bias and low CVs, highlighting their robustness to pre‐analytical variation.

**TABLE 4:** Bias and coefficient of variation (CV) between the routine centrifugation method and the high‐speed centrifugation method.

	Protocol A		Protocol B	
	Bias (yellow—green)	CV (%)	Bias (yellow—green)	CV (%)
Na	−0.31	3.34	3.21	5.97
K	0.4	84.67	0.93	84.71
Cl	−0.29	3.27	0.01	15.64
ALB	0	11.29	−0.02	13.39
CKMB	−0.45	47.16	2.76	68.8
HDL	1.61	17.36	1.75	19.02
UIBC	−4.51	24.4	2.62	23.47
PHO	−0.17	67.55	1.22	58.84
ALP	0.71	88.91	9.88	81.85
ALT	1.34	88.95	−2.92	84.16
IRON	−2.39	65.42	4.94	63.36
MG	0.02	14.94	−0.04	10.5
UA	0.09	19.83	−0.07	24.08
RF	−0.29	19.63	−0.44	18.92
DBIC	0	40.32	−0.04	58.08
AST	0.04	42.38	−0.11	41.18
FER	0.53	71.16	3.57	68.34
T. Br	0	44.44	−0.05	39.28
TP	−0.1	8.19	0.04	9.65
GGT	0.81	250.36	1.25	249.83
CHOL	4.25	16.57	5.16	14.54
CKN	−6.4	41.71	−3.57	40.13
GLU	4.15	58.07	10.1	62.28
TRIG	7.21	55.54	7.62	57.77
UREA	0.15	35.16	1.02	35.6
CA	−0.11	10.79	0.18	8.87
CRENZ	0.01	26.09	0.39	197.64
GLUB	0.08	16.86	0.12	22.1
LDL	3.35	20.7	−5.67	32.17
TIBC	−6.32	15.62	−1.93	15.35
FRT4	0.04	12.14	0.23	12.26
TSH	−0.26	73.82	−0.2	71.71
VITB12	0.89	82.84	−21.6	85.08
VITD	−3.84	57.85	−1.67	61.34

*Note:* Yellow tubes (no additive), green tube (lithium heparin).

Table 5 shows the centrifugation workload for 1000 daily samples under the routine centrifugation method (10 min) versus the high‐speed centrifugation method (5 min). For 1000 samples, the routine method requires 10,000 min of total centrifugation time, whereas the high‐speed method requires only 5000 min, effectively saving 5000 min—or 83.33 cumulative hours—per day. This represents a 50% reduction in total processing time. Such a significant efficiency gain enables laboratories to either double their daily sample throughput or complete existing workloads in half the time, thereby improving Turnaround Time (TAT). When combined with the previously demonstrated analytical accuracy, the high‐speed method proves to be a clinically safe and operationally superior alternative for high‐volume laboratories seeking to optimize performance.

**TABLE 5:** Turnaround Time (TAT) Simulation between the routine centrifugation method and the high‐speed centrifugation method.

Samples/day	Centrifuge time (A)	Centrifuge time (B)	Total time A (min)	Total time B (min)	Time saved (min)	Time saved (h)
1000	10 min	5 min	10,000	5000	5000	83.33

## 4 Discussion

Timely lab reporting is essential for effective clinical decisions with the turnaround time (TAT) which is heavily impacted by the pre‐analytical steps like centrifugation [9]. This study compared a standard centrifugation protocol with a shorter, high‐speed alternative (5 min at 4000 *g*). Notably, the results of this study show that the faster protocol maintains analytical accuracy for general chemistry, immunochemistry, and coagulation tests, while significantly reducing TAT. Thus, adopting this approach could streamline laboratory workflows, improve efficiency, and ensure rapid, reliable results without compromising diagnostic quality. Notably, the comparison between different analytes in this study revealed no statistically significant differences in the majority of analytes, indicating strong analytical agreement between the two protocols. Where statistical differences were noted, such as in sodium, ALT, CKMB, ALP, FRT4, PT, and INR, the magnitude of variation remained within clinically acceptable limits as defined by internal laboratory standards and CLIA guidelines. This supports the fact that these observed differences are statistically significant but not clinically relevant. Particularly for electrolytes like sodium, despite a small bias (*p* = 0.003), the values remained within reference ranges, confirming the suitability of the high‐speed centrifugation method for electrolyte panels. These findings are consistent with earlier studies, such as those by Tantisaranon et al., who concluded that changes in centrifugation time or force exert minimal clinical impact on most routine analytes when validated under quality control frameworks [3]. This study also included the Bland–Altman analysis, which further corroborates these findings. Notably, for the general chemistry analytes, data points were tightly clustered around zero bias, with the vast majority falling within the 95% limits of agreement. Notable exceptions, such as ALT and CKMB, exhibited mild downward biases (−1.91 and −4.5, respectively). However, these remained within permissible bias thresholds, and no values were identified as outliers. These results align with studies like Koenders et al., who emphasized that minor variations in ALT levels between centrifugation protocols did not affect the clinical categorization of liver function in most patients [10]. The observed biases in sodium, CKMB, and ALT may affect borderline clinical interpretations; however, values remained within diagnostic thresholds, which suggest that there is a minimal risk for misclassification, though caution is warranted in cases requiring high sensitivity, especially with tube‐related variability. The immunochemistry analytes also showed similar reliability. FRT4, TSH, and RF demonstrated negligible bias, while VITB12 and VITD showed minor but tolerable deviations. Importantly, CKMB retained acceptable limits of agreement. These findings resonate with those of Minder et al. [11], who observed that shorter centrifugation times did not affect immunoassay‐based analyte performance provided that no gel separators were used. This is especially relevant given that our study also employed non‐gel lithium heparin and serum tubes, minimizing analyte binding or phase separation issues. For coagulation analytes, the agreement between protocols was excellent. PT, INR, and APTT values showed minimal mean biases (all ≤ 0.2 units), and the spread was tightly controlled. This reinforces the conclusion that coagulation studies remain unaffected by reduced centrifugation times. These observations mirror findings by Chandler et al., who found that variations in centrifugation force had no meaningful and significant influence on INR or APTT when samples were processed promptly [12]. The bias and coefficient of variation (CV) analysis further support the high‐speed centrifugation method's precision and reproducibility. Several analytes demonstrated significant improvements in CV under the high‐speed centrifugation method, notably CRENZ (from 180.56% to 23.79%), DBIC, and potassium, suggesting enhanced precision with shorter centrifugation. High CVs were observed for a few analytes in both protocols, such as GGT and VITB12, but these were accompanied by stable mean biases, implying measurement consistency despite inherent biological or analytical variability. These findings reflect similar trends reported by Sönmez et al., who emphasized that centrifugation optimization can reduce pre‐analytical error and enhance reproducibility, especially for labile markers such as potassium and bilirubin fractions [13]. Interference indices, including lipemia, icterus, and hemolysis, remained unaffected or improved under the high‐speed centrifugation method. Notably, hemolysis rates decreased from 10.4% to 5.1%, which may be attributed to shorter centrifugation duration reducing red cell trauma. This is a valuable operational benefit, as hemolyzed samples frequently require recollection and delay result release. Similar reductions in hemolysis with modified centrifugation parameters have been reported by Allison et al., who found that optimizing rotor speed and duration can reduce red blood cell rupture, particularly in high‐throughput settings [14]. Moreover, the analytical reliability across both lithium heparin and serum tubes shows minimal bias for most analytes. Significant variation in CKMB, ALT, and CRENZ under faster centrifugation reflects tube‐dependent effects, aligning with Lima‐Oliveira et al., who noted that the tubes with different components and separators showed identical results in all parameters [11]. Notably, from an operational standpoint, this study shows the transformative impact of the high‐speed centrifugation method on TAT. For 1000 samples per day, the high‐speed centrifugation method reduced centrifugation time from 10,000 to 5000 min, effectively halving processing time and saving 83.33 cumulative hours. This highlights the lab's potential to either double its throughput or significantly reduce backlog and delays. This improvement is clinically meaningful, particularly in emergency departments or high‐volume central labs, where faster reporting directly correlates with improved patient care outcomes. Taken together, these findings support the adoption of the high‐speed centrifugation method as a clinically safe and operationally superior alternative. It maintains analytical accuracy across major test categories, reduces sample rejection due to hemolysis, and dramatically enhances workflow efficiency. Moreover, this study aligns well with current literature advocating for process streamlining in laboratory medicine, as highlighted in recommendations by the International Federation of Clinical Chemistry (IFCC), which emphasize optimizing pre‐analytical variables to support quality improvement [15].

Moreover, the recent international recommendations, which are particularly CLSI GP44‐A4 (2023), have increasingly emphasized the importance of validating centrifugation protocols across all analyte categories with a focus on preanalytical variability and clinical decision thresholds [16]. These organizations recommended that the laboratories should implement the standardized validation studies before adopting the non‐conventional centrifugation parameters, especially when diverging from the manufacturer recommendations. Our findings contribute to this effort by presenting the robust statistical comparisons and the Bland–Altman analyses to ensure the analytical equivalence. While our centrifugation protocol (5 min at 4000 *g*) diverges from the CLSI's traditional ranges, our findings align with the core principle of these guidelines, which is to ensure the analytical integrity without compromising the diagnostic interpretation. Moreover, there is a recent EFLM position paper that also supported the tailored preanalytical strategies in high‐throughput environments, provided that method‐specific validation is rigorously performed, which is a notion we addressed through comprehensive bias, CV, and agreement analyses [16].

There are several limitations of this study which include the use of non‐gel tubes only, which may not reflect performance with gel separators. The high‐speed centrifugation protocol (5 min at 4000 *g*) used in this study is experimental and not endorsed by major guidelines, requiring further validation. The real‐world TAT data were unavailable which limited the operational conclusions. The differences in analytes were not compared against the TEa limits due to lack of access. Additionally, while the analyte panel was comprehensive, not all the possible high‐sensitivity assays (e.g., troponin I, PTH) were evaluated. Future studies should examine protocol impact on a broader range of specialized biomarkers and assess long‐term instrument wear and maintenance associated with higher‐speed centrifugation.

## 5 Conclusion

This study concludes that the alternative high‐speed centrifugation provides comparable analytical accuracy to the standard method while significantly reducing turnaround time by half. Most analytes showed no clinically relevant differences, and even those with statistically significant variations remained within acceptable limits. Reduced hemolysis rates and improved precision in several parameters further support its adoption. The high‐speed centrifugation method offers a reliable, efficient, and safe solution for high‐throughput laboratories aiming to optimize workflow without compromising diagnostic quality.

## Conflicts of Interest

The authors declare no conflicts of interest.

## Data Availability

The data that support the findings of this study are available from the corresponding author upon reasonable request.
